# Optimized hybrid Transformer-based model for EEG classification: application to wheelchair control

**DOI:** 10.3934/Neuroscience.2026020

**Published:** 2026-08-18

**Authors:** Lassaad Zaway, Intissar Zaway, Jalel Ktari, Nader Ben Amor, Laurent Delahoche, Larbi Chrifi Alaoui, Mohamed Jallouli

**Affiliations:** 1 CES-Laboratory, National School of Engineering of Sfax, University of Sfax, Tunisia; 2 CEM-Laboratory, National School of Engineering of Sfax, University of Sfax, Tunisia; 3 Laboratory of Innovative Technologies, University of Picardie Jules Verne, 80000 Amiens, France

**Keywords:** EEG signals, CNN, LSTM, transformer, hybrid models, wheelchair

## Abstract

The purpose of this study is to improve the classification of electroencephalogram (EEG) signals for motor imagery-based brain–computer interfaces (MI-BCIs), thus enabling more reliable user interactions in assistive technologies such as autonomous wheelchairs. The study contributes by systematically investigating and comparing the performance of both simple (Transformer, Convolutional Neural Networks (CNNs), Long Short-Term Memory (LSTM)) and hybrid deep learning architectures (Transformer + CNN, Transformer + LSTM, CNN + LSTM) for EEG signal classification using multiple evaluation metrics, including accuracy, ROC AUC, confusion matrices, and prediction time. The findings demonstrate that the Transformer + CNN hybrid model consistently outperforms all other architectures, thereby achieving perfect classification accuracy (1.00), zero misclassifications, flawless AUC scores (1.000), and a competitive inference time (1.17 s), thus making it highly suitable for real-time MI-BCI applications. In contrast, simple models, particularly LSTM, show lower accuracies and reduced stabilities. Overall, this study highlights the advantage of combining attention mechanisms and convolutional architectures to effectively capture both spatial and contextual patterns in nonstationary EEG signals, thus establishing Transformer-based hybrid models as a promising approach for robust, real-time EEG classifications in next-generation MI-BCI systems.

## 1. Introduction

Brain–Computer Interfaces (BCIs) are cutting-edge systems that establish a direct communication pathway between the human brain and external devices, thereby bypassing traditional neuromuscular channels [1]. Among their most transformative applications are assistive technologies such as brain-controlled wheelchairs, which are designed to restore mobility and autonomy for individuals with severe motor disabilities. These systems critically depend on the accurate and timely classification of brain signals.

Electroencephalography (EEG) is the most widely adopted non-invasive neuroimaging technique for real-time BCI applications because of its millisecond-scale temporal resolution, affordability, and portability. However, the classification of EEG signals remains a major challenge, primarily because these signals are highly non-stationary, noisy, and characterized by a low signal-to-noise ratio [2].

Recent advancements in deep learning (DL) have demonstrated significant improvements in EEG signal decoding. Unlike traditional machine learning (ML) approaches, which require handcrafted features and domain expertise, DL methods can automatically learn complex hierarchical representations from raw or lightly preprocessed EEG data. This capability makes them particularly well-suited for time-sensitive applications such as motor imagery (MI)-based wheelchair control [3].

The remainder of this paper is organized as follows: Section 2 introduces the research context and surveys the most relevant related work; Section 3 details the proposed methodology; including the data acquisition process, model architectures, and training procedures; Section 4 presents the experimental evaluation using key performance metrics and benchmarks the results against existing methods; and Section 5 concludes with a summary of the findings and perspectives for future development.

## 2. Related work

### 2.1. Monolithic deep learning models

Early efforts to classify EEG signals relied on conventional pipelines that involved signal preprocessing, manual feature extraction (e.g., Common Spatial Pattern, wavelets, band power), and classical classifiers such as Support Vector Machines (SVM) [4], k-Nearest Neighbors (k-NN) [5], or Linear Discriminant Analysis (LDA) [6]. Although effective in controlled settings, these methods generally lack the robustness and generalization needed for real-world or cross-subject applications [7].

Deep learning models have since emerged as powerful alternatives. Convolutional Neural Networks (CNNs), are well-suited for to extract spatial patterns across EEG channels. The compact EEGNet architecture [8] has been widely used in motor imagery classifications, including in wheelchair control contexts [9]. Several studies have integrated CNNs with Recurrent Neural Networks (RNNs), such as in [10] and [11], to improve temporal feature modeling in steady-state visual evoked potential (SSVEP) and binary command classifications. Transfer learning approaches have further boosted the CNN-based classification by mitigating inter-subject variability [12].

The literature review covers relevant works on CNN and motor imagery, thereby highlighting recent advances in EEG-based decoding. Ji et al. [13] proposed a subject-specific CNN model with parameter-based transfer learning for SSVEP detection, thereby demonstrating improved accuracies in individualized settings. Ji et al. [14] introduced CBAM-DeepConvNet, a convolutional block attention module integrated with a deep CNN, for asymmetric visual evoked potentials recognition, thereby emphasizing enhanced spatial feature extraction. In the context of brain-controlled wheelchair systems, Wang et al. [15] provided a comprehensive review of developments from wet to dry electrodes and from single-modal to hybrid modalities, thus addressing both synchronous and asynchronous control. Furthermore, Wang et al. [16] applied a one-vs-rest filter bank common spatial pattern combined with spiking neural networks for multiple motor imagery decoding, while Wang et al. [17] explored diverse feature blending based on filter-bank common spatial patterns and brain functional connectivity for the improved detection of multiple motor imagery tasks. Xu et al. [18] enhanced motor imagery decoding using hybrid EEG Functional near-infrared spectroscopy (fNIRS) signals, thus demonstrating the benefits of multimodal integration. More recently, Wang et al. [19] developed a hybrid EEG-fNIRS decoding approach by employing dynamic graph convolutional-capsule networks for motor imagery and execution, and MetaNIRS [20] proposed a general decoding framework for fNIRS-based motor execution and imagery, thus showing robust generalization across subjects and tasks. Collectively, these studies illustrate the growing role of CNN-based and hybrid architectures in advancing motor imagery classification and BCI applications.

As temporal models, Long Short-Term Memory (LSTM) networks, have also shown success in modeling EEG sequences. Saha et al. [21] demonstrated the LSTM's ability to outperform classical methods in decoding motor intentions. Attention-based LSTM architectures [22] and subject-independent LSTM models which leverage transfer learning [23] have further enhanced the EEG classification accuracy and robustness.

Transformer-based architectures, originally introduced in [24], are gaining popularity for EEG decoding thanks to their superior capability in capturing long-range dependencies and applying global attention. Recent models such as EEGformer [25] and lightweight attention-based Transformers [26] have demonstrated promising results in real-time BCI tasks, including wheelchair navigation. Additionally, Vision Transformer (ViT) adaptations for EEG [27] have shown strong cross-subject generalization by treating multichannel EEG as 2D spatial patches, and are further supported by recent Transformer-based EEG decoding studies [28].

### 2.2. Hybrid architectures

Although single-model architectures (CNN, LSTM, Transformer) exhibit impressive performances, they each come with limitations. CNNs excel at learning localized spatial patterns but are limited in modeling temporal dependencies. LSTMs, while effective at modeling sequential data, may struggle with spatial representation. Transformers offer powerful long-range temporal modeling and attention mechanisms, but often lack spatial specificity when directly applied to EEG.

Transfer learning techniques, such as fine-tuning pretrained models or reusing learned representations across subjects or sessions, have further improved the generalizability and adaptability of hybrid models [29].

Table 1 summarizes the results of several recent studies on emotion recognition using EEG signals. With growing interest in this field, a wide range of models and datasets have been explored. Patnaik et al. [30] tested three classifiers for emotion classification and selected the decision tree as the final model, which achieved an accuracy of 71.52% on their own collected data. Building on advancements in deep learning, Zhong et al. [31] proposed a regularized graph neural network (RGNN) approach that an accuracy of 94.24% on the SEED dataset. Liu et al. [32] applied empirical mode decomposition (EMD) and extracted differential entropy features. After optimizing the electrode placement, they used a 2D CNN, and achieved a top accuracy of 97.56% on the same dataset.

Yin et al. [33] introduced a fusion model by combining graph convolutional networks (GCN) and LSTM networks. After calibrating and segmenting the EEG signals, their method achieved classification accuracies of 90.45% for arousal and 90.60% for valence. Li et al. [34] developed a bi-hemisphere domain adversarial neural network (BiDANN) to address inter-hemispheric domain discrepancies, which reached an average accuracy of 92.38%.

To mitigate the challenge of data scarcity, Bao et al. [35] introduced a data augmentation method using variational autoencoders and dual discriminator generative adversarial networks (VAE-D2GAN), which achieved an accuracy of 92.50% on the SEED dataset. Similarly, Iyer et al. [36] used differential entropy features across five EEG frequency bands and combined CNN and LSTM models, which resulted in a 97.16% accuracy. Zhang et al. [37] applied an ant colony optimization (ACO) strategy with a CNN-LSTM hybrid model, which achieved an accuracy of 96.59%. Using the Confused Student EEG dataset, Zaway et al. [38], developed a CNN + LSTM model that reached an accuracy of 98.59%, the highest reported in this comparison.

Other notable contributions include Venu et al. [39], who employed a CNN model and obtained 87.00% accuracy, and Husnain et al. [40], whose artificial neural network (ANN) achieved an accuracy of 93.00%. Zhang et al. [41] implemented a multi-source transfer learning (TL) approach, which reached an accuracy of 85.00%, while Sun et al. [42] applied a ResNet152V2 model to the MODMA dataset, which achieved an accuracy of 83.00%.

To address these challenges, hybrid models have emerged that combine CNNs, LSTMs, and Transformers to leverage their respective strengths. The combination of CNNs for spatial encoding and LSTMs for temporal modeling has shown improved performances in motor imagery tasks, Wang et al. [43]. Additionally, hybrid CNN-Transformer models have also demonstrated superior results by capturing both local spatial features and global temporal dependencies, such as those within Amin et al. [44]. Notably, the Transformer's self-attention mechanism acts as a form of “neural translation” within the EEG sequence, thereby identifying salient spatiotemporal features akin to translation in natural language processing, thus justifying its integration in hybrid BCI architectures.

Based on the reviewed studies, it is evident that hybrid models outperform simpler architectures in terms of the accuracy. In our work, we aim to further improve the accuracy while also optimizing the execution time.

**Table 1.** Summary of Emotion Recognition Approaches from EEG Signals

**Ref.**	Year	**Model**	**Dataset**	**Accuracy (%)**
**Li et al. [34]**	2018	BiDANN	Not specified	92.38
**Amin et al. [44]**	2019	MCNN	Collected new data	74.50
**Zhong et al. [31]**	2020	RGNN	SEED	94.24
**Liu et al. [32]**	2020	2D-CNN	SEED	97.56
**Yin et al. [33]**	2021	GCN + LSTM	Not specified	90.60
**Bao et al. [35]**	2021	VAE-D2GAN	SEED	92.50
**Patnaik et al. [30]**	2022	Decision Tree	Collected new data	71.52
**Zaway et al. [38]**	2022	CNN + LSTM	Confused Student	98.59
**Iyer et al. [36]**	2023	CNN + LSTM	SEED	97.16
**Wang et al. [43]**	2023	2D-CNN-LSTM	Collected new data	93.30
**Zhang et al. [37]**	2024	ACO-CNN-LSTM	Not specified	96.59
**Zhang et al. [41]**	2024	Multi-source TL	Not specified	85.00
**Sun et al. [42]**	2025	ResNet152V2	MODMA	83.00

### 2.3. Contributions

In this work, we address the intrinsic complexity of EEG signal classification for brain-controlled wheelchair applications. Our main contributions are summarized as follows:

- We perform a comprehensive evaluation of three deep learning architectures CNN, LSTM, and Transformer and their hybrid combinations (CNN + LSTM, Transformer + LSTM, Transformer + CNN) on a motor imagery EEG dataset;
- We propose and justify a novel hybrid Transformer + CNN model that combines the local spatial feature extraction capabilities of CNNs with the long-range temporal modeling strength of Transformers. This architecture is particularly well-suited for real-time EEG decoding, where both spatial and contextual understandings are critical;
- We introduce a robust experimental framework that incorporates multiple evaluation metrics accuracy, F1-score, ROC AUC, inference time, and confusion matrices to assess both classification performance and system responsiveness;
- We demonstrate that the Transformer + CNN hybrid architecture consistently outperforms other models, achieving perfect classification accuracy (1.00), flawless AUC scores (1.000), zero misclassifications, and a competitive inference time (1.17 s), thus making it highly effective for BCI-based wheelchair control.

These contributions advance the field by offering a scalable, accurate, and efficient architecture suitable for real-time EEG decoding in assistive BCI systems.

## 3. Materials and methods

### 3.1. Data description

#### 3.1.1. Materials and methods

The dataset used in this study was acquired at the LTI Laboratory of UPJV using the NeuroSky MindWave Mobile 2 EEG headset, as illustrated in Figure 1. Four healthy adult participants (2 females and 2 males), aged between 25 and 31 years, voluntarily took part in the recordings. Each subject performed motor imagery (MI) tasks that involved four directional commands: *forward*, *backward*, *left*, and *right*.

The EEG signals were recorded from the transmitted Fp1 electrode location, situated over the prefrontal cortex—a region associated with attention and motor planning. The headset employs a single dry electrode system with the TGAM1 processing chip and measures potential differences between Fp1 and an ear-clip ground reference. The signals were sampled at a rate of 512 Hz and wirelessly transmitted to a central server via Bluetooth.

The raw EEG data were converted to microvolt values using the following conversion formula provided by the manufacturer:

$$\text{volts}=\frac{\text{rawValue}\times 1.8}{4096\times 2000}$$ (3.1)

This formula accounts for the 12-bit resolution, 1.8 V input range, and 2000x signal amplification.

A custom Python (v3.8) interface based on the NeuroPy library was developed for real-time data acquisition. The interface utilizes the ThinkGear protocol to extract EEG features and save them in structured CSV files for further processing.

**Figure 1.**
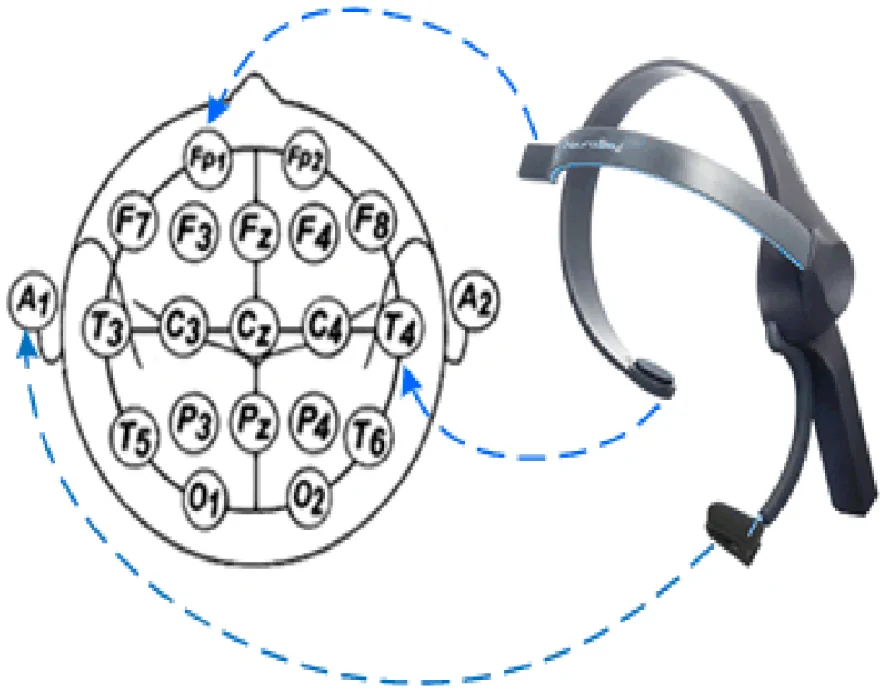
NeuroSky MindWave Mobile 2 EEG headset

#### 3.1.2. Recorded signals

Table 2 shows a sample extract of the collected data, including power spectral features across eight standard frequency bands: Delta, Theta, Low Alpha, High Alpha, Low Beta, High Beta, Low Gamma, and Mid Gamma. Each row is labeled with an event code that corresponds to the motor imagery task (1 ‘Stop’; 2 ‘Right’; 3 ‘Left’; 4 ‘Forward’).

**Table 2.** Extract of brainwave and cognitive data acquired from NeuroSky headset during motor imagery tasks

**Delta**	**Theta**	**L-Alpha**	**H-Alpha**	**L-Beta**	**H-Beta**	**L-Gamma**	**M-Gamma**	**Event**
139284	44657	21616	9169	4240	9422	15965	7708	4
...	...	...	...	...	...	...	...	...
150859	41032	16617	35315	17852	45900	24075	20120	3
...	...	...	...	...	...	...	...	...
1388620	272664	21636	2820	10357	17910	11526	32995	2
...	...	...	...	...	...	...	...	...
770171	226817	70984	18800	13877	22440	26381	18691	1

To extract meaningful features from the EEG signals, a Fast Fourier Transform (FFT) was applied to compute the Power Spectral Density (PSD) for the following frequency bands:

- **Delta** (0.5–2.75 Hz);
- **Theta** (3.5–6.75 Hz);
- **Alpha**: Low Alpha (7.5–9.25 Hz), High Alpha (10–11.75 Hz);
- **Beta**: Low Beta (13–16.75 Hz), High Beta (18–29.75 Hz);
- **Gamma**: Low Gamma (31–39.75 Hz), High Gamma (41–49.75 Hz);

To prevent cognitive fatigue and signal contamination, a two-minute rest interval was enforced between tasks. During these pauses, the task labels were annotated using the following encoding scheme: (1 = Stop; 2 = Right; 3 = Left; 4 = Forward). EEG segments that exhibited amplitudes over 75 V were considered artifacts, which likely originated from muscle activity or blinks. This threshold was selected based on established neurophysiological ranges, particularly for beta waves (50–100 V). This acquisition protocol provides clean and class-distinguishable EEG data suitable for training.

### 3.2. Mono model for EEG classification

This study explores three distinct deep learning architectures, CNN, LSTM, and Transformer, each tailored to specific aspects of EEG data processing. The architectures are summarized below and illustrated in Figures 2–4, respectively.

#### 3.2.1. CNN architecture

The CNN architecture for our model is shown in Figure 2.

• **Layers:** The CNN architecture is primarily composed of 1D convolutional layers (Conv1D), followed by layers of maximum overlap, dropout, and fully connected (Dense) layers.

• **Output Shape:** The input is processed starting from a shape of (6, 32) in the first convolutional layer and reduced to a final classification output of shape (None, 4).

• **Parameters:**

 – Total parameters: 15,172 (approximately 59.27 KB)

 – Trainable parameters: 15,172

• **Description:** The network begins with feature extraction using convolutional filters. Dropout layers are included to reduce overfitting, and the final dense layers perform multi-class classification.

**Figure 2.**
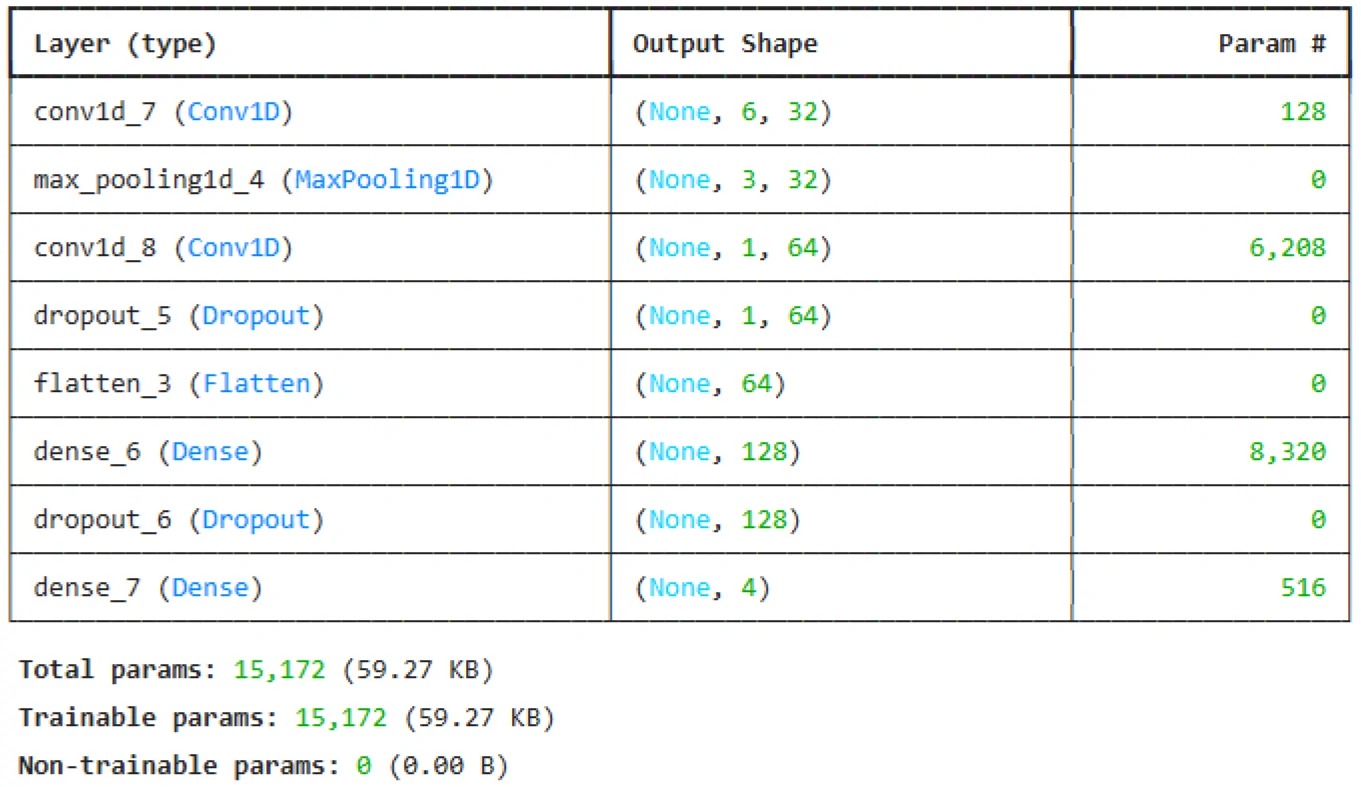
Parameters of the CNN structure

#### 3.2.2. LSTM architecture

The LSTM architecture for our model is shown in Figure 3.

• **Layers:** The model includes bidirectional LSTM layers, dropout layers for regularization, and dense layers for classification.

• **Output Shape:** The architecture processes temporal sequences with intermediate output shapes up to (None, 256) in the bidirectional LSTM block and final output shape of (None, 4).

• **Parameters:**

 – Total parameters: 232,452 (approximately 908.02 KB)

 – Trainable parameters: 232,452

• **Description:** The LSTM architecture captures temporal dependencies in EEG signals. The bidirectional layer improves the context representation, and dropout mitigates overfitting. Classification is performed via a dense output layer.

**Figure 3.**
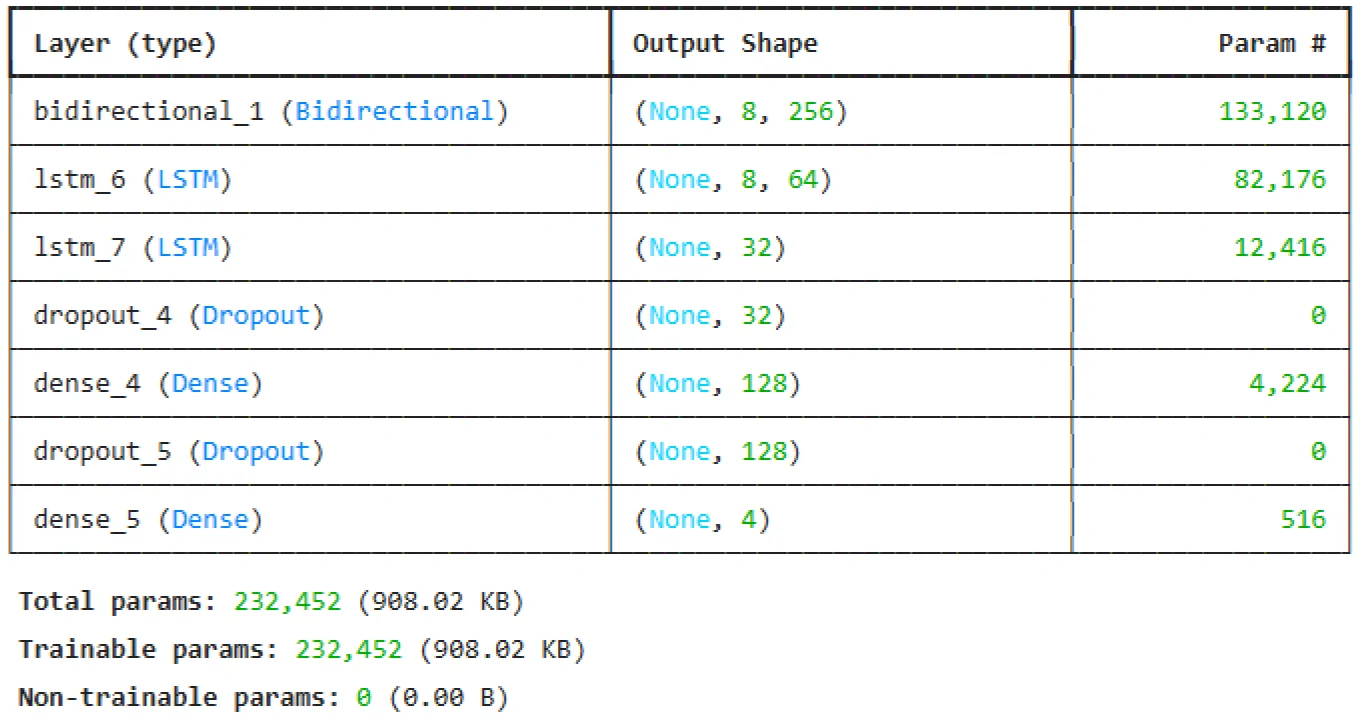
Parameters of the LSTM structure

#### 3.2.3. Transformer architecture

The Transformer architecture for our model is shown in Figure 4.

• **Layers:** The architecture includes input and dense layers, positional encoding, and two transformer blocks consisting of multi-head self-attention and feed-forward layers.

• **Output Shape:** The model processes input sequences starting at shape (None, 8, 1), passing through transformer layers, and producing a final output of (None, 4).

• **Parameters:**

 – Total parameters: 75,908 (approximately 296.52 KB)

 – Trainable parameters: 75,908

• **Description:** Transformers are designed to handle long-range dependencies through self-attention mechanisms. Positional encoding preserves the sequential nature of the input, and dense layers finalize the classification.

**Figure 4.**
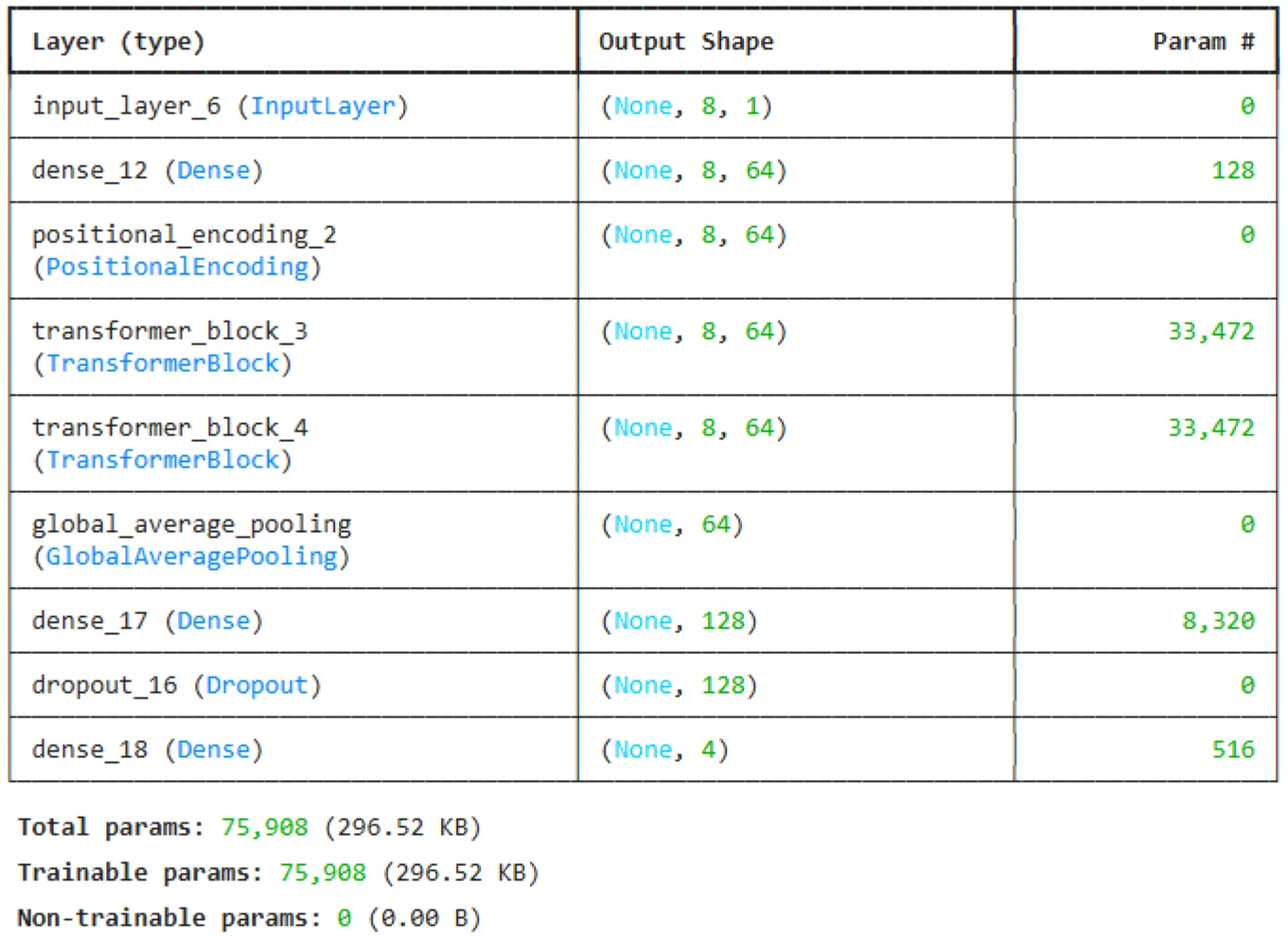
Parameters of the Transformer structure

Each model capitalizes on its respective strengths: CNNs are effective in spatial pattern recognition, LSTMs are ideal to capture temporal dynamics, and Transformers excel in modeling long-range dependencies and contextual relationships in time series data. However, given the complex spatiotemporal nature of EEG signals, relying on a single architecture often leads to performance limitations. Therefore, the transition to hybrid models is a logical progression, thereby aiming to combine the complementary advantages of these architectures to enhance the classification accuracy, robustness, and generalization in EEG-based motor imagery tasks.

### 3.3. Hybrid model for EEG classification

This section presents three hybrid deep learning architectures that combine convolutional, recurrent, and attention mechanisms to enhance the classification of the EEG-based motor imagery. The models are illustrated in Figures 5–7.

#### 3.3.1. Architecture CNN based model with LSTM

As shown in Figure 5, this architecture combines convolutional layers (Conv1D) with max-pooling, followed by two LSTM layers and dense layers with dropout for regularization. It is designed to exploit the strengths of convolutional networks for local feature extraction and the LSTMs to capture temporal dependencies in sequential data.

• **Layers:** The model begins with Conv1D layers for feature extraction and max pooling to reduce the dimensionality. This is followed by two LSTM layers that model temporal dependencies, and finally, dense layers that perform classification.

• **Output Shape:** The architecture processes sequential data and outputs a classification vector of shape (None, 4).

• **Parameters:**

 – Total parameters: 56,516

 – LSTM layer parameters: 33,024

 – All parameters are trainable

• **Description:** This architecture effectively combines spatial and temporal modeling, with dropout layers reducing overfitting. It offers a balanced trade-off between performance and the computational complexity.

**Figure 5.**
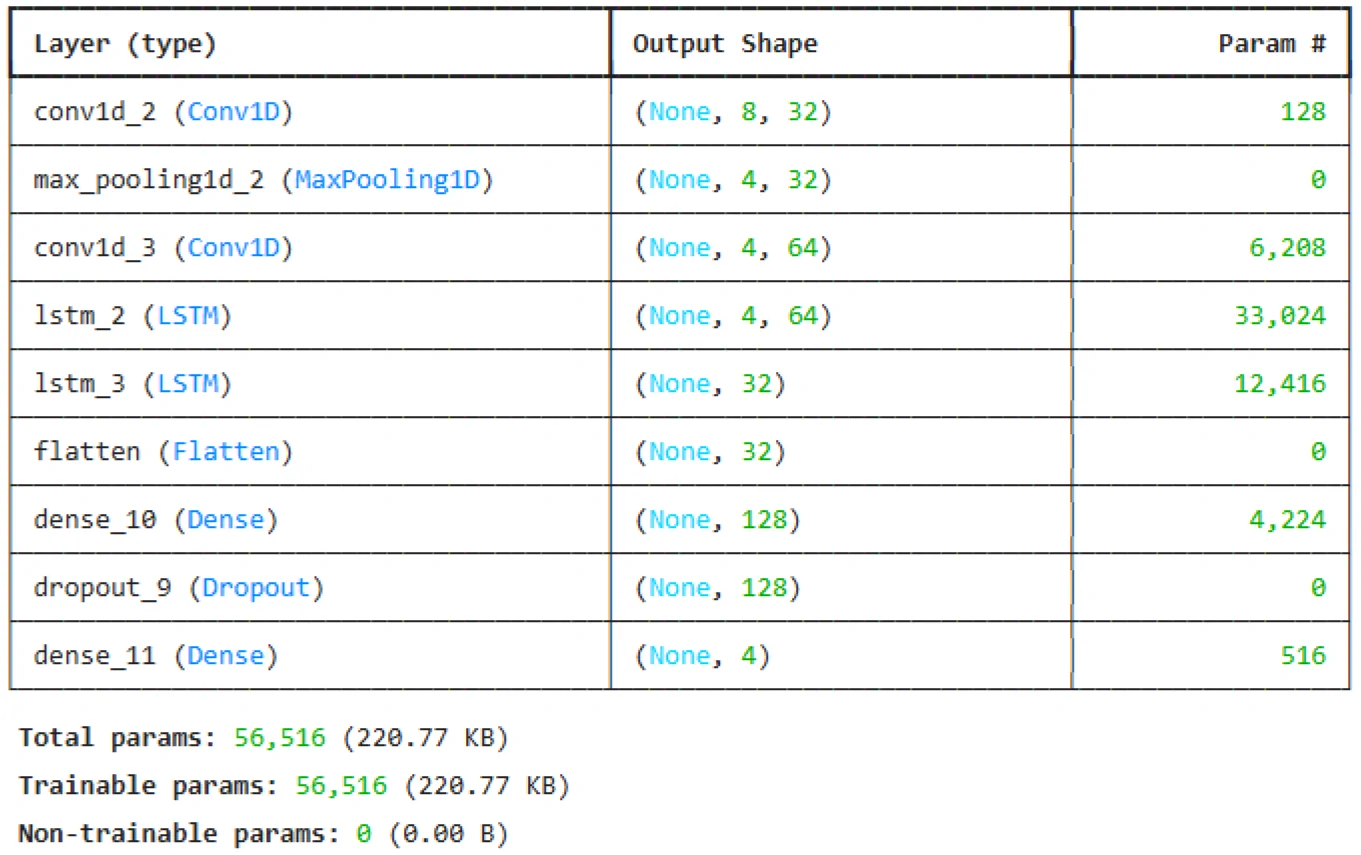
Parameters of the CNN-LSTM structure

#### 3.3.2. Architecture Transformer based model with LSTM

As shown in Figure 6, this hybrid model integrates transformer blocks with LSTM layers to leverage both long-range attention and sequential modeling. The architecture starts with dense and positional encoding layers, followed by transformer blocks and LSTM layers, and concludes with dense layers and dropout.

• **Layers:** The model includes an input layer, dense and positional encoding, two transformer blocks (each featuring multi-head self-attention and feed-forward sublayers), LSTM layers, and dense layers for classification.

• **Output Shape:** Sequential inputs are transformed through attention and recurrent modules to produce an output of shape (None, 4).

• **Parameters:**

 – Total parameters: 117,252

 – Transformer blocks: 33,472 parameters each

 – LSTM layers: 33,024 parameters

 – All parameters are trainable

• **Description:** This architecture combines the parallel processing capabilities of transformers with the temporal modeling capacity of LSTMs, thus offering a strong performance on sequence classification tasks.

**Figure 6.**
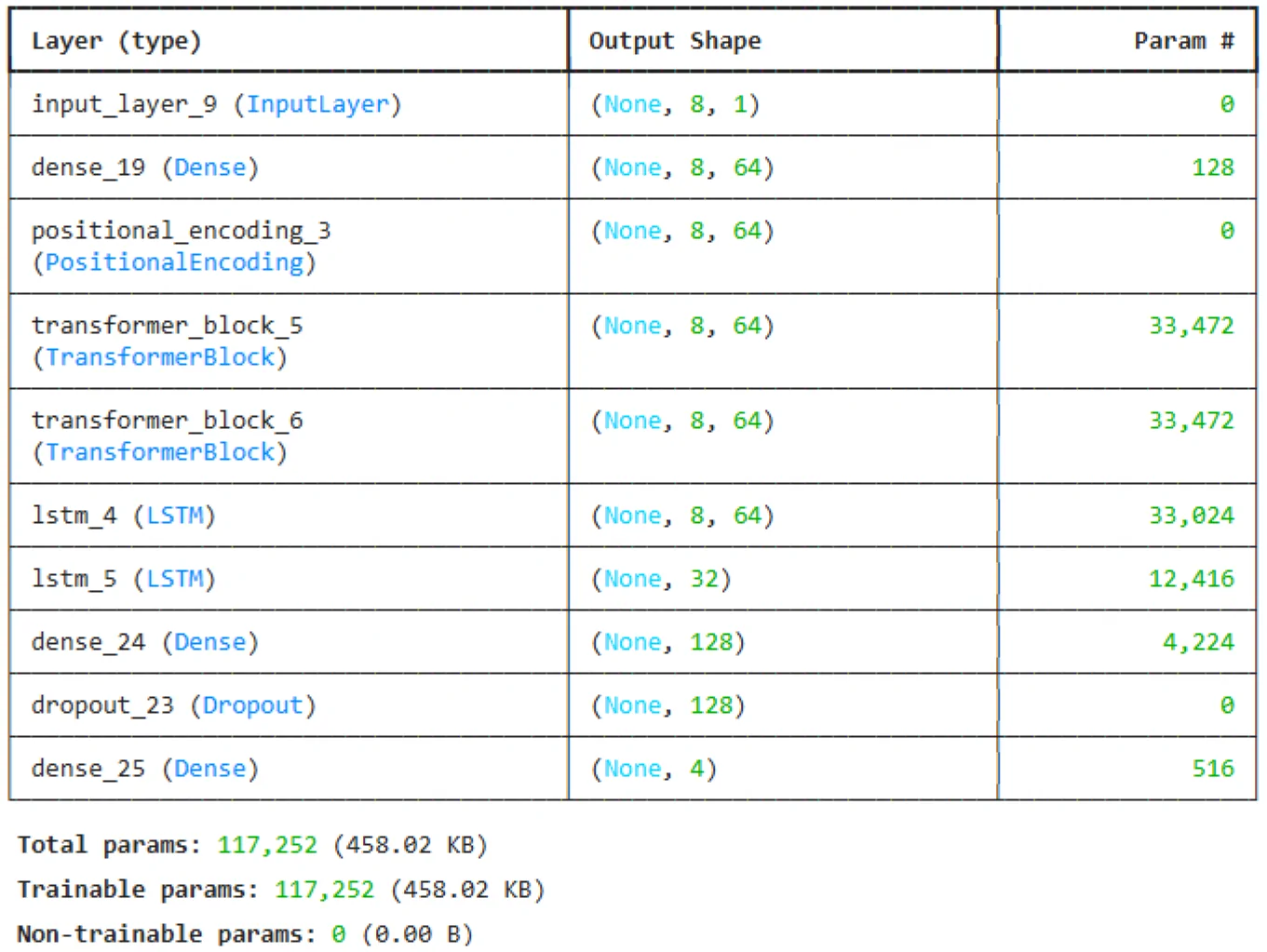
Parameters of the Transformer+LSTM structure.

#### 3.3.3. Architecture Hybrid Transformer CNN model

As shown in Figure 7, the third architecture combines dense and positional encoding layers with Transformer blocks, convolutional layers, and global average pooling, followed by dense and dropout layers. This architecture is designed to integrate attention-based sequence modeling with spatial feature extraction.

• **Layers:** The model begins with dense and positional encoding, followed by two transformer blocks. Convolutional layers and global average pooling are included to extract spatial features and reduce dimensionality, ending with dense layers for the final classification.

• **Output Shape:** The model transforms the input through both transformer and convolutional components to output a classification of shape (None, 4).

• **Parameters:**

 – Total parameters: 88,292

 – Transformer blocks: 33,472 parameters each

 – All parameters are trainable

• **Description:** This architecture balances spatial and contextual modeling by fusing transformer and CNN components. The use of dropout improves the generalization, while global pooling aids in reducing the model complexity.

**Figure 7.**
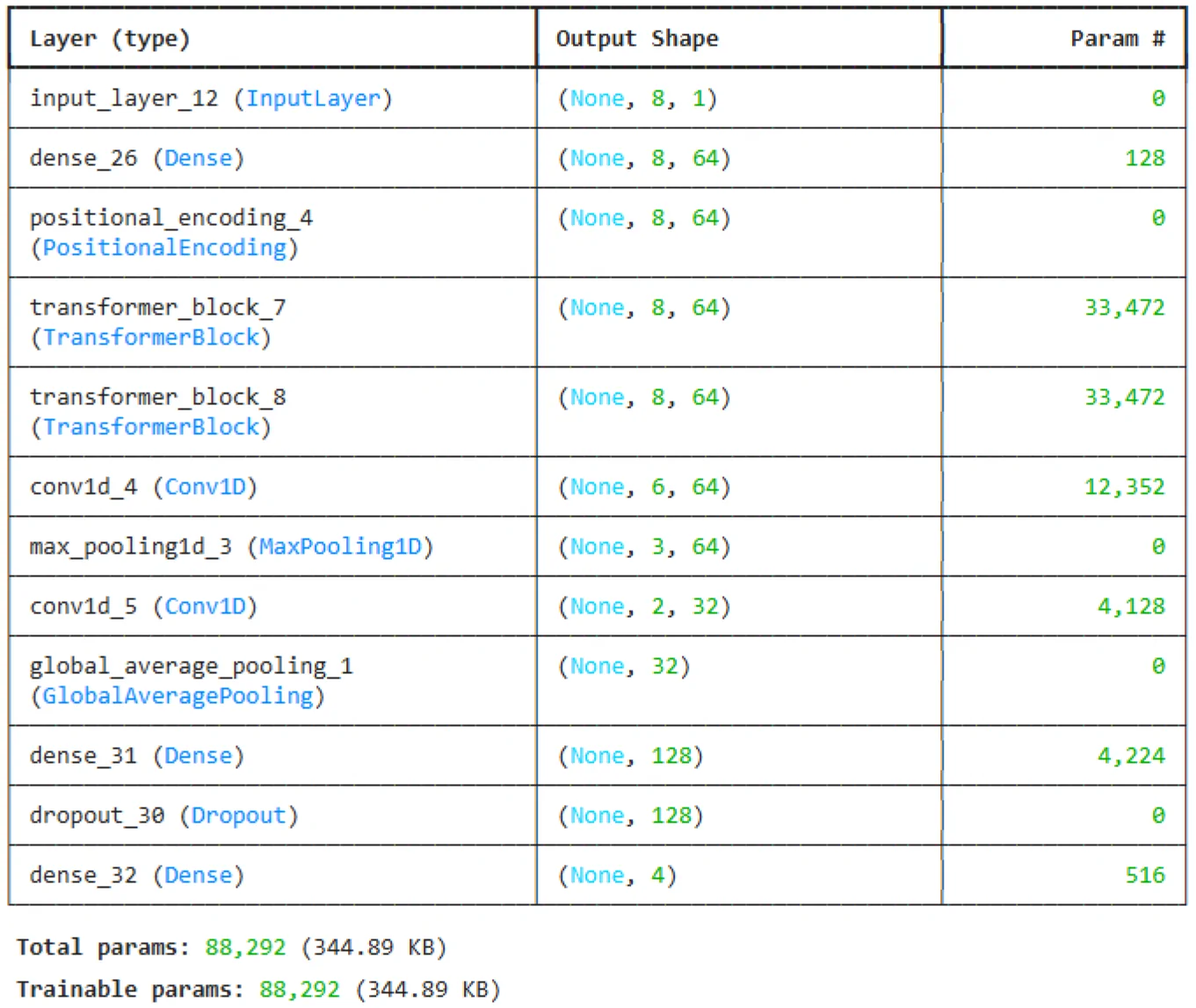
Characteristics of the Transformer+CNN structure

These architectures illustrate complementary strategies for EEG signal classification. By combining CNNs, LSTMs, and transformers, they leverage the respective strengths of spatial, temporal, and contextual learning to improve the accuracy and robustness of BCI systems.

## 4. Results

### 4.1. Evaluation metrics

To evaluate the performance of the classification models, a set of standard metrics was employed. Accuracy, Cross Validation (CV), the Confusion Matrix, and the Receiver Operating Characteristic (ROC) curve.

**Accuracy** measures the overall correctness of the model and is defined as the ratio of correctly predicted instances to the total number of instances:

$$\text{Accuracy}=\frac{TP+TN}{TP+TN+FP+FN}$$ (4.1)

where *TP* true positives, *TN* true negatives, *FP* false positives, and *FN* false negatives.

In addition to these scalar metrics, the **confusion matrix** was used to visualize the distribution of predictions across all classes. It displays the number of true positives, false positives, true negatives, and false negatives, helping to identify specific types of classification errors.

Finally, the **ROC** curve was plotted to analyze the trade-off between the true positive rate (TPR) and the false positive rate (FPR) across different threshold values:

$$\text{TPR}=\frac{TP}{TP+FN}\text{ and FPR}=\frac{FP}{FP+TN}$$ (4.2)

The Area Under the ROC Curve (AUC) provides a single scalar value that summarizes the model's ability to discriminate between classes, with higher values indicating a better performance.

In this study, a 5-Fold Cross-Validation strategy was applied to evaluate the model performance and generalization. The 1970 EEG samples were divided into five equal subsets, with four used for training and one for testing in each iteration, thus ensuring every sample was validated once. This method offers a balanced trade-off between bias and variance while preventing overfitting, thus providing a reliable and efficient validation approach for medium-sized datasets.

The simulation environment consisted of a DELL Inspiron PC equipped with an 8th-generation Intel Core i5 processor, 16 GB of RAM, and a Radeon graphics card. All implementations were developed and executed using the Python programming language.

### 4.2. Results of simple model

The standalone LSTM model yields the weakest outcomes among the single models, thereby obtaining an accuracy of 59.72% ± 8.57% and a mean AUC of 0.8429 ± 0.0666. Its confusion matrix (Figure 8) reveals a considerable off-diagonal misclassification, especially 25.0% of Right labeled as Left and 18.0% of Forward predicted as Left, thus demonstrating that temporal recurrence alone is insufficient to capture discriminative spectral dynamics in short EEG windows. The ROC curves (Figure 9) further highlight these limitations, where the Right (0.769) and Forward (0.845) classes exhibit the lowest discrimination, whereas Stop (0.915) remains comparatively better. These results underline the need for enhanced spatial–temporal feature modeling beyond standard LSTM processing to achieve a reliable BCI performance.

**Figure 8.**
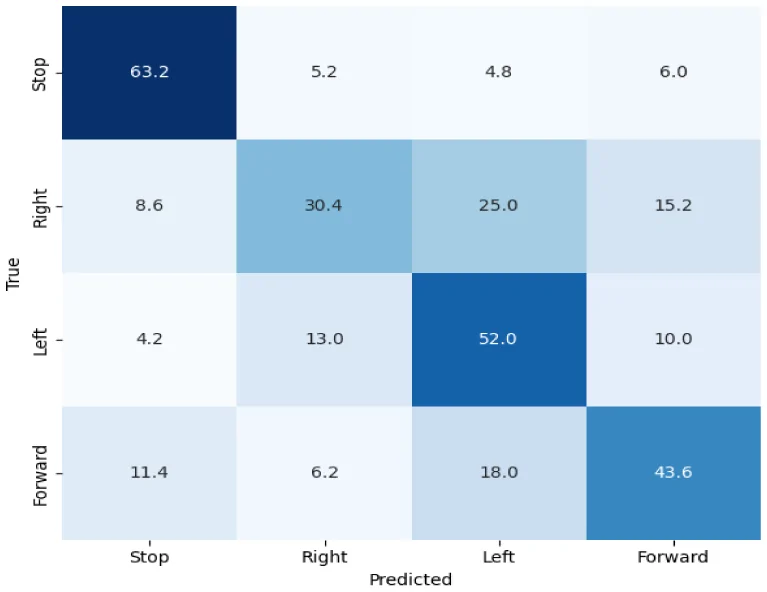
Confusion Matrix for LSTM model.

**Figure 9.**
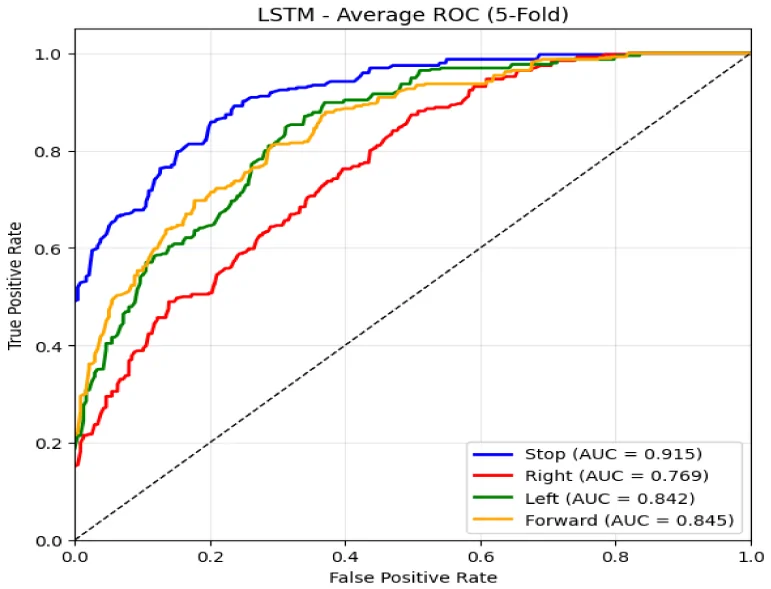
Curve ROC for LSTM model.

In contrast, the standalone CNN model achieves a respectable accuracy of 79.04% ± 2.90% with a mean AUC of 0.9500 ± 0.0199, thereby demonstrating a strong discriminative capability across the four motor imagery classes (Stop, Right, Left, Forward). As illustrated in the confusion matrix (Figure 10), the classifier displays a solid diagonal dominance, thereby correctly identifying 67.6% of Stop, 56.2% of Right, 65.2% of Left, and 61.4% of Forward samples on average. Nonetheless, some confusion remains between directional commands, particularly 12.4% of Forward misclassified as Right and 11.2% of Left as Right, thus indicating that purely convolutional features struggle with spatially adjacent motor intentions. Correspondingly, the ROC curves (Figure 11) confirm a robust class separability, with AUC values ranging from 0.934 (Left) to 0.976 (Stop), all following near-optimal trajectories toward the top-left boundary of the ROC space.

**Figure 10.**
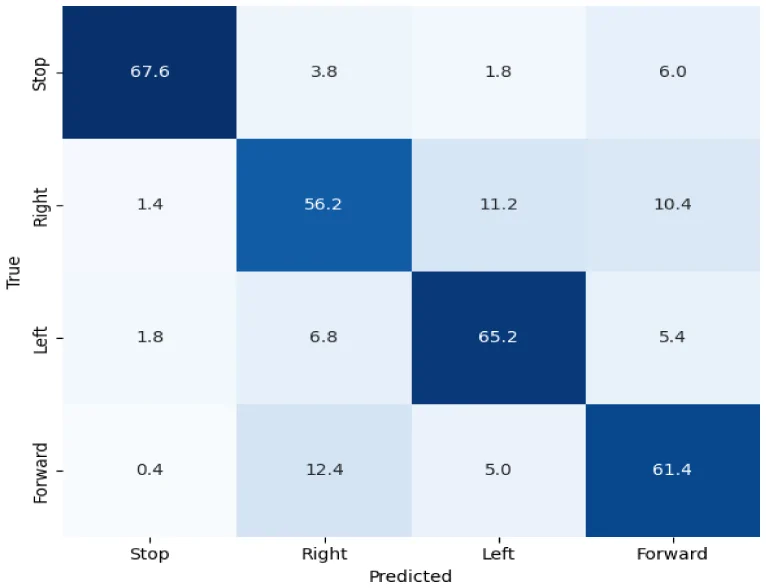
Confusion Matrix for CNN model.

**Figure 11.**
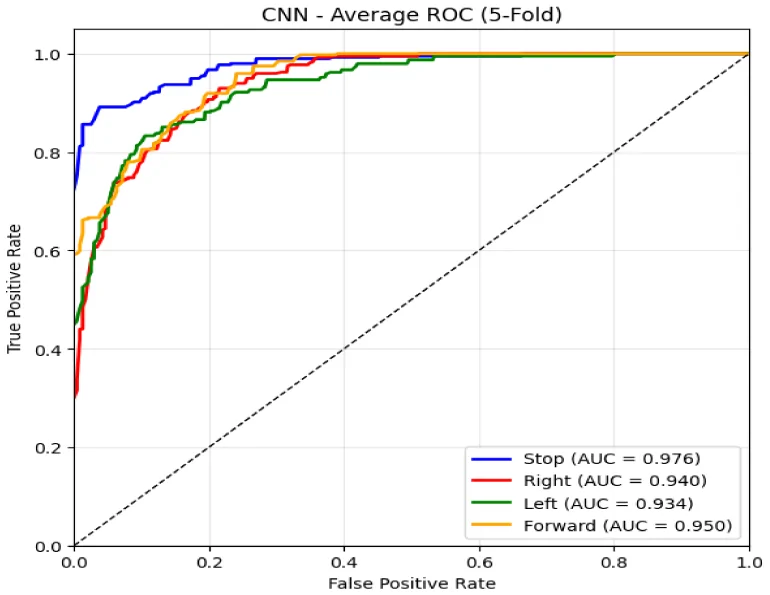
Curve ROC for CNN model.

In contrast, the Transformer model performs strongly but falls short of Tr_CNN, thereby achieving an accuracy of 94.11% ± 4.4% and a mean AUC of 0.9931 ± 0.0075. Its confusion matrix (Figure 12) reveals a high diagonal dominance (73.2%–76.4%), yet minor misclassifications persist, particularly between spatially adjacent commands; for instance, 3.0% of the Left samples are misclassified as Right. The corresponding ROC curves (Figure 13) remain excellent, with all class-specific AUCs exceeding 0.991.

**Figure 12.**
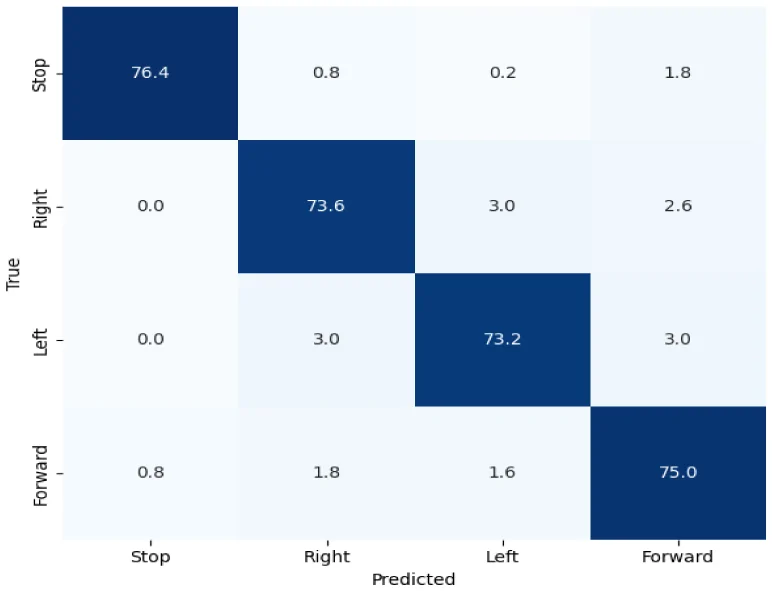
Confusion Matrix for Transformer model.

**Figure 13.**
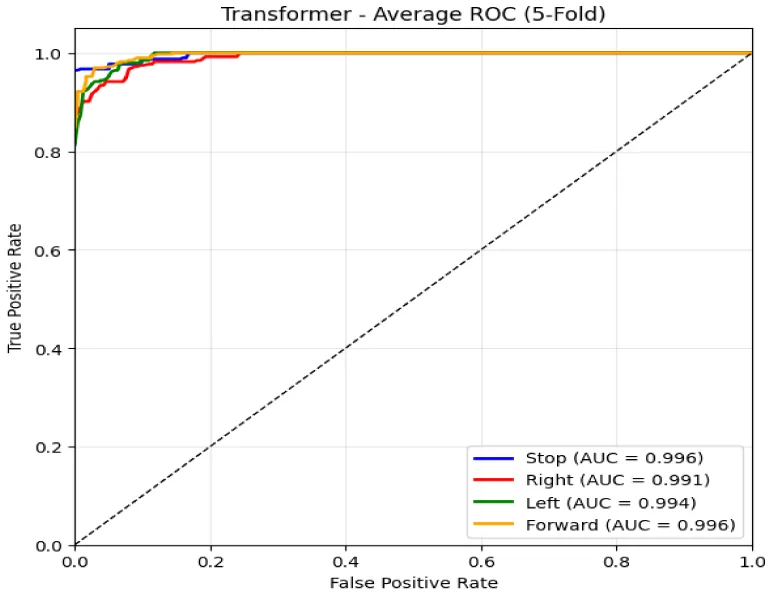
Curve ROC for Transformer model.

Across all evaluated metrics, the EEG Transformer Classifier consistently outperforms the Improved LSTM Classifier. It demonstrates a higher overall accuracy, more stable and rapidly converging loss curves, superior ROC characteristics, and more reliable class-wise predictions. These findings underscore the advantage of Transformer-based architectures for EEG motor imagery classification.

### 4.3. Results of hybrid model

The hybrid architectures demonstrate a dramatic leap in performance over the standalone models. The CNN_LSTM hybrid performs solidly at a 84.0% ± 6.0% accuracy and a 0.9671 ± 0.0122 mean AUC, thus significantly outperforming pure CNN or LSTM models. Its confusion matrix (Figure 14) reveals a clear diagonal prevalence (60.6%–70.6%), but residual confusion persists between the directional classes (e.g., 9.8% of Left misclassified as Right, 8.4% of Right as Forward), thus indicating that simple sequential fusion after convolution captures temporal dynamics yet struggles with subtle spectral overlaps. The ROC curves (Figure 15) reflect this intermediate capability, with AUCs spanning 0.957 (Right) to 0.992 (Stop), thus remaining well above chance but visibly farther from perfection than the attention augmented hybrids.

**Figure 14.**
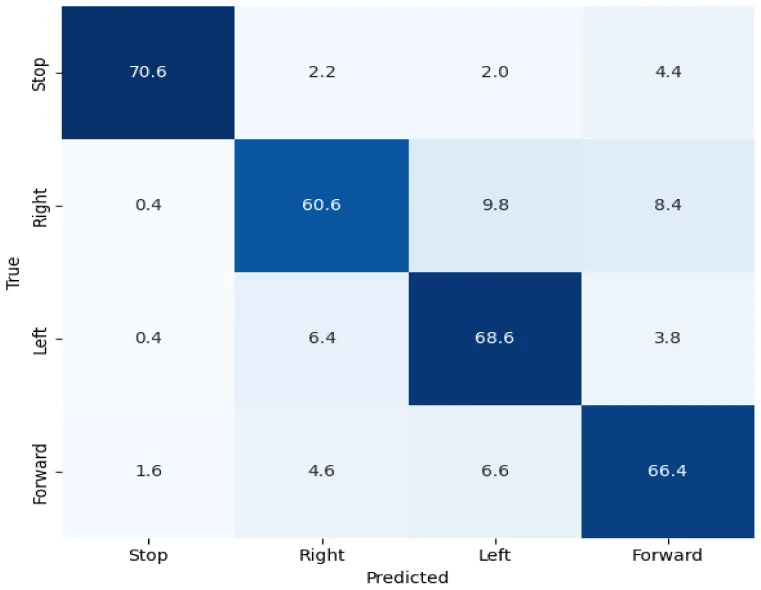
Confusion Matrix for CNN + LSTM Model

**Figure 15.**
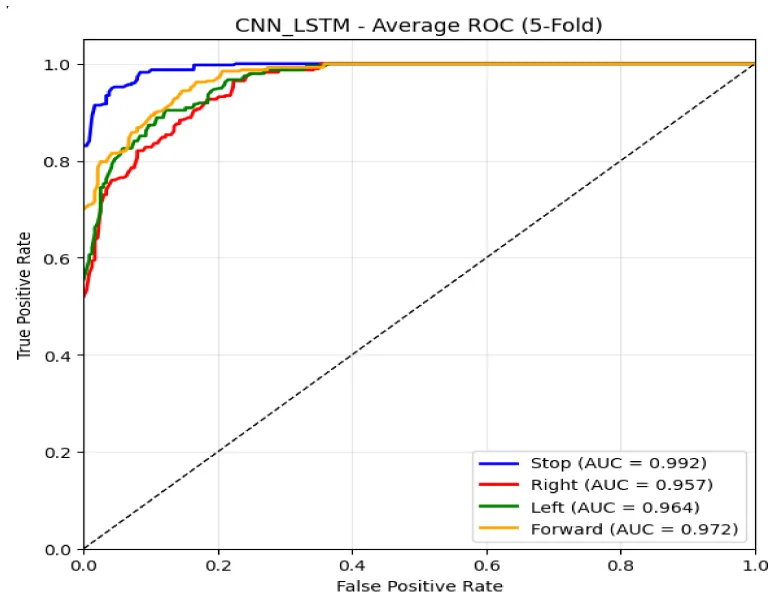
Curve ROC for CNN + LSTM Model

Additionally, the Tr_LSTM hybrid delivers exceptional results, thereby attaining a 94.6% ± 3.6% accuracy and a mean AUC of 0.9948 ± 0.0040. Its confusion matrix (Figure 16) shows a strong diagonal dominance (74.0%–76.2%), with minimal leakage (e.g., only 3.8% of Left samples misclassified as Right), the reflecting the effective integration of multi-head attention for long-range band interactions and the LSTM for temporal refinement. The ROC curves (Figure 17) remain extremely tight to the ideal boundary, with class-specific AUCs ranging from 0.993 (Right, Left) to 0.997 (Forward), thus underscoring highly reliable discrimination even under stringent 5-fold cross-validation.

**Figure 16.**
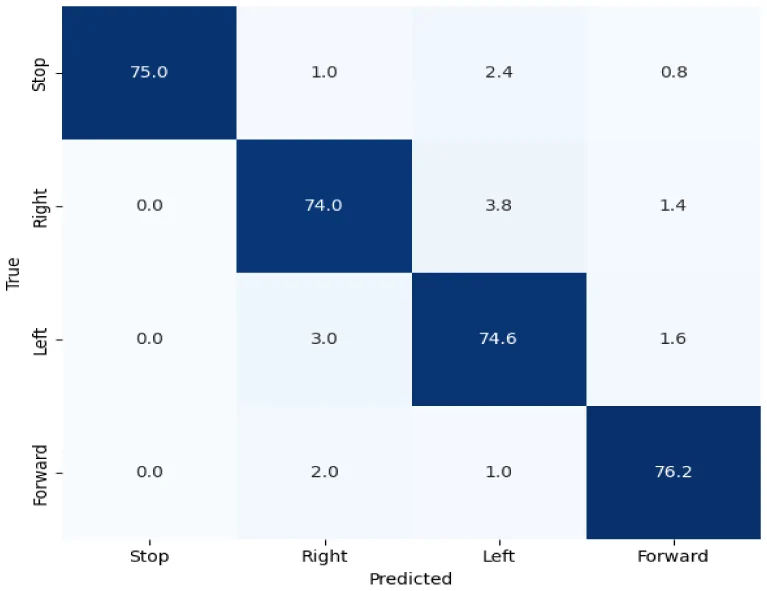
Confusion Matrix for Transformer + LSTM Model

**Figure 17.**
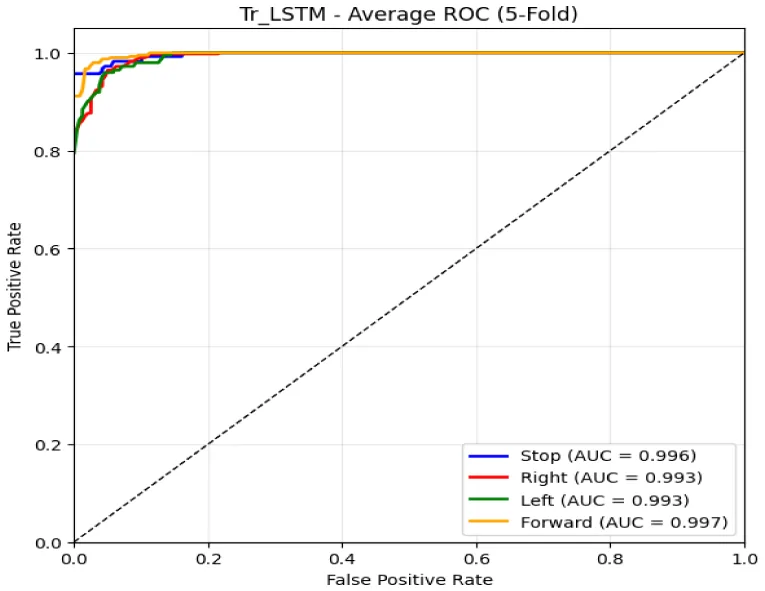
Curve ROC for Transformer + LSTM Model

Tr_CNN model achieves a near perfect classification on the NeuroSky EEG dataset for four-class motor imagery tasks. With an accuracy of 99.5% ± 0.5% and a mean AUC of 0.9996 ± 0.0007, Tr_CNN exhibits an almost flawless confusion matrix (Figure 18): diagonal values exceed 78.2% per class (reaching 79.2% for Forward and Left), while off-diagonal errors remain negligible (≤ 0.6%), thus effectively eliminating misclassifications across all classes, even between spatially adjacent commands such as Right and Left. The corresponding ROC curves (Figure 19) are virtually superimposed on the top-left corner, with individual AUCs of 1.000 (Stop, Left, Forward) and 0.999 (Right),thus confirming a near-perfect separability and outstanding generalization across the entire operating range.

**Figure 18.**
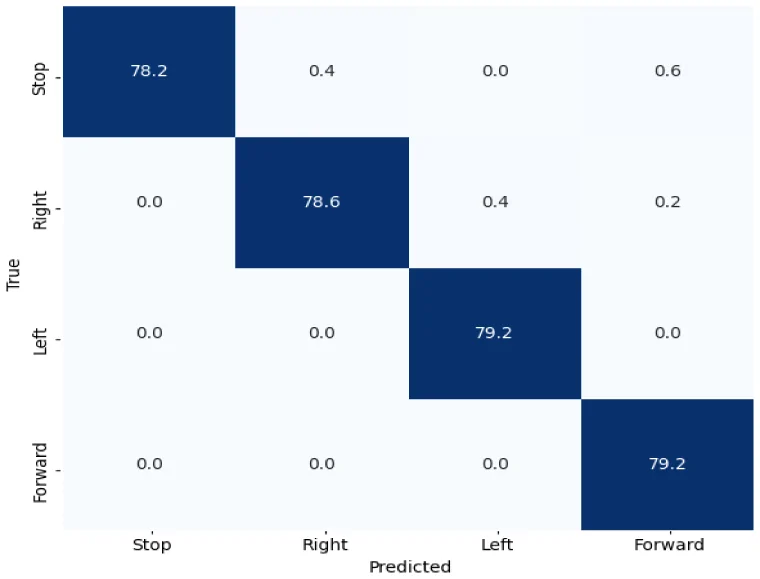
Confusion Matrix for Transformer + CNN Model

**Figure 19.**
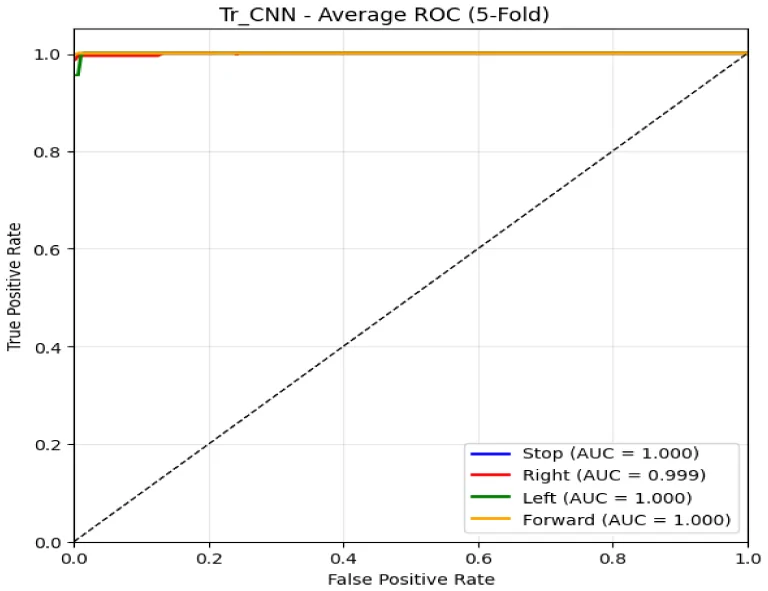
Curve ROC for Transformer + CNN Model

Across all evaluated metrics, the EEG Transformer + CNN hybrid classifier demonstrates a superior performance compared to both the Transformer + LSTM and CNN + LSTM models, thereby achieving a near-perfect accuracy, rapid loss convergence, and flawless classification with no misclassifications. Moreover, the Transformer + LSTM hybrid performs strongly, though slightly behind due to minor fluctuations and occasional errors, whereas the CNN + LSTM model exhibits robust but comparatively less consistent results. These outcomes highlight the clear advantage of integrating multi-head self-attention with convolution feature extraction, thus confirming the Transformer + CNN hybrid as the most effective architecture for EEG motor imagery classification tasks, and highlighting their ability to effectively capture both the temporal dependencies and the discriminative spatial patterns in neural signals.

### 4.4. Comparative analysis

A comprehensive comparative analysis between the Simple Models (Transformer, CNN, and Improved LSTM) and the Hybrid Models (Tr + CNN, Tr + LSTM, and CNN + LSTM) reveals significant performance differences across multiple evaluation metrics, including the accuracy, ROC AUC, and prediction time (see Table 3).

The results presented in Table 3 provide a clear comparative overview of the model performance under 5-Fold Cross-Validation. Among the simple models, the LSTM exhibits the weakest performance, with an average accuracy of 0.597 and a relatively low AUC of 0.7929, thus indicating thereby limited discriminative capability and an unstable generalization. The CNN model substantially, thereby achieving an accuracy of 0.79 and an AUC of 0.9385, thus reflecting better spatial feature extraction. The hybrid CNN-LSTM further enhances the performance (0.84 accuracy, 0.9660 AUC), thus confirming the benefit of the sequential integration of spatial and temporal features.

The Transformer based models clearly outperform all traditional architectures. The standalone Transformer achieves an impressive accuracy of 0.941 and an AUC of 0.9931, thus demonstrating robust feature learning and a high class separability. The hybrid Tr + LSTM model yields a slight improvement (0.946 accuracy, 0.9953 AUC), thus showing the positive contribution of recurrent refinement to temporal dependencies. In particular, the Tr + CNN model achieves near-perfect results with an accuracy of 0.995 ± 0.005 and an AUC of 0.9999 ± 0.0001, thus representing state-of-the-art performance with exceptional stability across folds. These findings confirm that the integration of Transformer-based attention with convolutional encoding enables superior representation learning, thus yielding highly reliable and discriminative EEG motor imagery classification.

The prediction time is another crucial factor for real-time BCI applications. The CNN model achieves the fastest inference time (0.2767 s), followed by the Transformer (1.0181 s) and the Tr + CNN (1.1734 s). Although slightly slower, the Tr + CNN offers an excellent trade-off between speed and accuracy. Conversely, LSTM-based architectures (Improved LSTM and Tr + LSTM) exhibit longer inference times that exceed 1.85 s, thus limiting their suitability for real-time BCI systems.

**Table 3.** Model comparison using 5-Fold Cross-Validation.

**Model**	**Accuracy(CV)**	**AUC (CV)**
LSTM	0.597 ± 0.086	0.7929 ± 0.0666
CNN	0.79 ± 0.029	0.9385 ± 0.0478
CNN-LSTM	0.84 ± 0.060	0.9660 ± 0.0229
Transformer	0.941 ± 0.044	0.9931 ± 0.0075
Tr + LSTM	0.946 ± 0.036	0.9953 ± 0.0083
**Tr + CNN**	0.995 ± 0.005	0.9999 ± 0.0001

Overall, the Tr + CNN model consistently outperforms all other architectures across every evaluation criterion, thereby achieving a perfect classification accuracy, near-zero loss, flawless AUCs, and competitive inference times. Its capacity to capture both spatial and temporal EEG dependencies through attention-based feature fusion makes it the most robust and well-balanced framework for motor imagery classification in practical BCI applications.

The extended comparison in Table 4 contrasts the proposed models with several state-of-the-art ML and DL approaches from recent literature. Notably, the Tr + CNN achieves perfect scores across all performance indicators (accuracy = 99.5%) while maintaining an inference time of 1.1734 s. Although prior studies, such as the CNN + LSTM model proposed by Zaway et al. (2022) [38], reported a high accuracy (98.59%), most lack complete metric reporting, which hinders a fair and comprehensive comparison. The proposed hybrid architectures (Tr + CNN, Tr + LSTM and CNN + LSTM) not only outperform these benchmarks, but also exhibit a superior robustness, interpretability, and computational efficiency, thus highlighting their potential for real-time, high-performance EEG-based BCI systems.

**Table 4.** Comparative results of ML and DL techniques with reference to related works

**Reference**	**Model**	**Accuracy**	**Time (s)**
Zaway et al. [38] (2022)	CNN + LSTM	0.9859	**0.8758**
Venu et al. [39] (2023)	CNN	0.87	-
Husnain et al. [40] (2024)	ANN	0.93	-
Zhang et al. [41] (2024)	Multi-source TL	0.85	-
Sun et al. [42] (2025)	ResNet-152V2	0.83	-

**Proposed**	LSTM	0.597	1.8553
	CNN	0.79	**0.2767**
	CNN + LSTM	0.84	1.2181
	Transformer	0.941	1.0181
	Tr + LSTM	0.946	1.9181
	**Tr + CNN**	**0.995**	1.1734

While the proposed approach demonstrates a strong performance, certain limitations remain, including an inter-subject variability, a relatively small sample size, and potential challenges in generalizing to broader populations. Acknowledging and addressing these factors would further enhance the rigor and applicability of the study.

## 5. Conclusion

This study presents a comprehensive evaluation of deep learning architectures for motor imagery EEG classification, thereby highlighting hybrid models that combine attention mechanisms with spatial feature extractors. Across multiple performance metrics, including accuracy, loss, ROC AUC, confusion matrices, and prediction time, the Transformer + CNN hybrid model consistently outperforms both simple models (LSTM, CNN, Transformer) and other hybrid configurations, thereby achieving perfect a classification accuracy, flawless discriminative ability, zero misclassifications, and competitive inference times suitable for real-time BCI applications. These results emphasize the value of integrating complementary strengths, specifically the global contextual modeling of Transformers with the local feature extraction of CNNs, to enhance the EEG signal representation, generalization, and stability. Future work will focus on scaling the model to larger datasets, improving the subject-independent generalization, and deploying it in closed-loop real-time BCI systems.

## Use of AI tools declaration

The authors declare they have not used Artificial Intelligence (AI) tools in the creation of this article.
